# The Impact of the Socio-Demographic Characteristics of Complementary and Alternative Medicine Users in Serbia on OTC Drug Consumption

**DOI:** 10.3389/fpubh.2019.00303

**Published:** 2019-10-24

**Authors:** Marina Luketina-Sunjka, Nemanja Rancic, Natasa Mihailovic, Mihajlo Jakovljevic

**Affiliations:** ^1^European Center for Peace and Development, University for Peace established by the United Nations, Belgrade, Serbia; ^2^Faculty of Medicine of the Military Medical Academy, Centre for Clinical Pharmacology, University of Defense, Belgrade, Serbia; ^3^Department of Biostatistics and Informatics, Institute of Public Health Kragujevac, Kragujevac, Serbia; ^4^Division of Health Economics, Lund University, Lund, Sweden

**Keywords:** CAM, complementary and alternative medicine, OTC drugs, socio-demographic factors, national health survey, health care

## Abstract

The Aim of this research is to analyze how the socio-demographic characteristics of users of Complementary and Alternative Medicine (CAM) in Serbia influence and impact their consumption of OTC drugs. Respondents and methods: The study employed the third edition of the National Health Survey of the Republic of Serbia, published in 2013, as a data source covering the Serbian population. The sample comprised of 550 interviewed individuals who had been applying a variety of CAM treatments over the previous 12 months. Their socio-demographic characteristics were used as independent variables impacting the consumption of OTC drugs over the previous 2-week period, representing the dependent variable.

**Results:** Two thirds (65.3%) of the CAM users consumed OTC drugs at their own discretion, without recommendation by a physician or a relevant prescription. Users of OTC drugs were most often females whose ages ranged within the average interval of 49.16 ± 16.02, whose education level was to secondary school diploma, who were married and employed, lived in urban areas, mostly Belgrade, belonged to the middle-income group, and followed relevant headlines via public information channels (TV, the internet, radio, and print). Comparison of the results revealed, on the one hand, that 2/3 of respondents who had used and 1/3 of those who did not consume OTC drugs had undergone fecal occult blood tests over the past year (*p* < 0.05) and, on the other hand, that those respondents had been less frequently hospitalized in the previous year (*p* = 0.05). In addition, the same responders were found to access available health care services more frequently than did others (*p* < 0.05).

**Conclusion:** Since, according to the statistics, it is highly likely that respondents who were CAM- and OTC drug-users would be less frequently hospitalized and not use medical leave, these results provide a strong indication that this phenomenon should be investigated in more depth. Moreover, the areas to be considered when defining strategies for determining patient treatments should also include the influence of socio-demographic factors on the patient's consciousness that would enable easier understanding of the proper usage of OTC drugs.

## Introduction

Complementary and Alternative Medicine (CAM) has attracted global interest in both developed and underdeveloped countries (1). During the past several decades, post-industrial societies have been characterized to have a “blooming” prevalence and incidence of so-called “prosperity diseases,” i.e., chronic non-infectious diseases. In 2016, the WHO reported that out of 56.9 million deaths worldwide, one-third were caused by ischemic heart disease and stroke. In the past 15 years, cardiovascular diseases have been determined to be the leading cause of death globally (2). Moreover, chronic non-infectious diseases have also been recognized as causing the majority of deaths in Serbia (3). This phenomenon has seriously put to the test the availability and limits of so-called Conventional or Science Medicine. Facing such long-term challenges, in May 2009, the WHO Assembly adopted Resolution 62.13, which calls on all member states and governments to cooperate and share knowledge while simultaneously working on strengthening bonds between conventional and traditional practitioners (4). In most Balkan countries, the introduction of legislation for the development of medicine was interrupted by the civil war that took place in the 90s (5), and this continued through the country's transitional period (6). CAM development also suffered: only in 2007, rather late when compared to other European countries, did the Ministry of Health of the Republic of Serbia enable the introduction of Complementary and Alternative Medicine, to be practiced by health workers only (7).

The abbreviation “OTC” stands for “Over the Counter,” and such drugs are medicines that require no prescription. Research shows that 81% of adults in the USA practice taking OTC drugs as a **first** reaction to a minor health condition (8). A household in the USA, on average, spends about 442 USD on OTC drugs per year (9). As per the results of a systematic review covering 27 studies aiming to calculate the proportion of CAM users within the population of cardiovascular patients, the prevalence of CAM consumption was recorded to be between 22 and 68%; 2–46% consumed herbal medicines, 3–54% consumed vitamins, minerals, and other dietetic supplements (mostly Vitamin B12 or Vitamin B complex, Vitamin C, Vitamin E, Chondroitin Glucosamine, Coenzyme Q10, Calcium, and Magnesium) (10).

Globally, CAM budgets vary significantly, and comparisons are complex firstly due to the different currencies used, secondly because there is no standardization, causing CAM definitions and categorizations to differ, and thirdly because different research time intervals have been used (11). For these reasons, the comparison of OTC use data among populations is complicated.

Based on a National Population-Based Survey in Australia conducted on CAM users, it was noted that 621 million AUD (12) was paid for CAM out-of-pocket in 1993, 1671 million AUD in 2000 (13), 1308 million AUD in 2004 (14), and 1860 million AUD in 2005 (15). In 1990, US citizens spent 10.3 billion USD out of their own pockets (16, 17), in 1997 they spent 34.4 billion USD (18, 19), in 2007, 33.9 billion USD (20, 21), and in 2012, 30.2 billion USD (22). In the UK, a systematic review of studies researching CAM usage prevalence determined that monthly expenses per patient amounted to 15.99 GBP (23). In 1999, the estimated expenses reached 1.6 billion GBP in the UK (24).

The Serbian health care system is funded by both public finances and private contributions (25). About 69% of total current health expenditure is financed by public sources, and more than 90% of public sources are financed through the Republican Health Insurance Fund (26, 27), with supplementary funding from budgetary sources (the Ministry of Finance Fund for the Unemployed, the Pension Fund, etc.) Private funding is completely based on out-of-pocket payments (28), and it is supplemented by contributions from companies that fund their own institutions that specialize in the treatment of occupational diseases and provide primary care services.

The aim of this particular research was to determine which factors impact the usage of OTC drugs among the CAM-using population, or in other words, besides considering health conditions, financial status, lifestyle, the presence of chronic illnesses, and other factors have been included in our observations, as these may contribute to the occurrence of simultaneous usage of CAM and OTC drugs.

## Materials and Methods

The most recent health research data on the population of the Republic of Serbia were used for this research. It is from a cross-sectional study run by the Ministry of Health of the Republic of Serbia throughout 2013.

In the research, interviews were used, and a horizontal approach was taken, enabling the collection of more personalized data from a single individual (health condition, whether they accessed health care services, habits, and lifestyle). The questions and indicators used in the questionnaires were standardized to those adopted by the European Union, in addition to using indicators standardized to those in the “Health For All” database of the WHO or recommended to become EU indicators (29, 30).

Surveys were conducted as a cross-sectional study. The population presented in the research included adults aged 19 and over. Individuals living in care institutions (elderly care homes, prisons, and psychiatric institutions) and in Kosovo and Metohija were not included in the research. The research used a national representation sample, which is a stratified two-phase sample without repetition. The sample frame in the research included all the households listed in censuse to 2011. In order to obtain a random sample, two techniques were used: stratification and multiple-phase sampling. Stratification was conducted in such a way that each of four geographical areas (Vojvodina, Belgrade, Sumadija and west Serbia, and south and east Serbia) represented one main stratum in the sample. Each stratum was divided into cities and other regions. The total number of strata was eight. Two-phase sampling included local communities, selected on the basis of probability proportional to their size, and households as units of the second phase selected on the basis of the linear sampling method with a random start and uniform selection steps. All respondents that had used CAM (acupuncture, homeopathy, phytotherapy/herbal therapy, or chiropractic) over the previous 12 months and were living in the Republic of Serbia were extracted from this database, totaling 550.

Individuals living in care institutions (elderly care homes, prisons, and psychiatric institutions) and in Kosovo and Metohija were not included in the research. The principles of ICH Good Clinical Practice were followed strictly, and approval was obtained from the Ethics Committee of the Republic of Serbia. The research used data from the Third National Health Survey of Serbia, sponsored by the Ministry of Health's Batut Institute, which provided data to the faculty for the purpose of additional research. The Ethical Standards for Healthcare Research are aligned with the International Medical Association Declaration of Helsinki and legislation specific to the laws of Serbia. Aiming to align with GDPR policies to preserve the discretion of the respondent data collected, all steps were adopted that are stipulated by the law on the protection of personal data (Official Gazette of the Republic of Serbia No. 97/08, 104/09), the Official Statistics Law (Official Gazette of the Republic of Serbia No. 104/09), and Directive 95/46/EC of the European Parliament on the protection of individuals with regard to the processing of personal data and on the free movement of such data (31).

### Statistical Methods

Continuous variables were presented as mean ± standard deviation, and categorical variables as frequency (n) and percentage (%). The Chi-square test, representing the univariant method, was used for categorial data, and the Students' *t-*test was used for continuous data. Factor analysis was applied as the multivariant technique. The following variables were analyzed through factor analysis: sex, age, education, region, accommodation, work status, well-being index, health self-evaluation, commitment to being healthy, record of chronic disease, medical leave, hospitalization, visits to day-care hospitals, home medical care and Emergency Room Assistance in the past 12 months, consumption of OTC drugs over the previous 2 weeks, risky behavior, routine checks and screening for colon cancer, and health care non-utilization (communalities higher than 0.2). All analyses were performed using SPSS, version 19.

## Results

In Serbia, two-thirds (65.3%) of CAM users consume OTC drugs at their own discretion, neither due to a physician's recommendation nor through prescription. OTC drug consumers are most frequently females with an average age of 49.16 ± 16.02, possessing a high-school diploma, married and employed, most frequently living in urban areas, mostly Belgrade, categorized as middle-income as per the “wellbeing index,” and who follow health topics via publicly available information (TV, the internet, radio, and print).

By comparing the basic socio-demographic characteristics of respondents that consume and that do no consume OTC drugs, it is evident that the consumers are mostly females (Chi-square test = 9.87, df = 1, *p* < 0.05) and that OTC consumers are, on average, 4.5 years older than those that do not use OTC drugs (t = −3.06, df = 548, *p* < 0.05).

There was no statistically significant difference in the use of analgesics with respect to gender, educational attainment, region and place of residence, self-evaluation of health status, presence of chronic health disorders, and long-term illness. On the other hand, individuals falling into the middle-income category more often consume analgesics at their own discretion (Chi-square test = 8.72, df = 2, *p* < 0.05), while this practice is rarest among respondents living in the Belgrade region (Chi-square test = 8.68, df = 3, *p* < 0.05).

The consumers of OTC drugs mostly evaluate themselves as being in good health, i.e., average, and, even though they are in the habit of risky behavior, have usually never undergone screening tests for colon cancer. Of those respondents, 56% suffer from chronic health disorders and visit health care institutions more frequently than others. Mostly, the users of OTC drugs had not taken any medical leave, been hospitalized, or used day-care hospitals, emergency assistance, or home medical care over the preceding 12 months (Table 1).

**Table 1:** Clinical characteristics and health care practices of participants.

**Characteristics**	**Users OTC n (%)**	**Non-OTC users n (%)**	***p*-value #**
**Self-evaluation of health**			
Very good	39 (58.2)	28 (41.8)	>0.05
Good	126 (64.3)	70 (35.7)	
Average	130 (695)	57 (30.5)	
Bad	49 (63.6)	28 (36.4)	
Very bad	15 (65.2)	8 (34.8)	
**Risky behavior**			
Yes	187 (67.8)	89 (32.2)	>0.05
No	167 (62.8)	99 (37.2)	
Already ill	5 (62.5)	3 (37.5)	
**Fecal occult blood tests**			
Over the previous year	16 (69.6)	7 (30.4)	<0.05
More than a year ago	24 (49.0)	25 (51.0)	
Never	317 (66.7)	158 (33.3)	
**Colonoscopy**			
Over the previous year	8 (66.7)	4 (33.3)	>0.05
More than a year ago	30 (57.7)	22 (42.3)	
Never	320 (66.1)	164 (33.9)	
Chronic disease^*^			
Yes	202 (66.9)	100 (33.10)	>0.05
No	157 (63.6)	90 (36.4)	
Medical leave^*^			
Yes	40 (66.7)	20 (33.3)	>0.05
No	92 (63.0)	54 (37.0)	
Hospitalization^*^			
Yes	41 (55.4)	33 (44.6)	<0.05
No	318 (66.8)	158 (33.2)	
**“Day patient”** ^*^			
Yes	53 (63.1)	31 (36.9)	>0.05
No	306 (65.7)	160 (34.3)	
Emergency assistance^*^			
Yes	37 (63.8)	21 (36.2)	>0.05
No	322 (65.4)	170 (34.6)	
Home medical care^*^			
Yes	7 (53.8)	6 (46.2)	>0.05
No	352 (65.5)	185 (34.5)	
**Health care non-utilization**			
Yes	162 (70.1)	69 (29.9)	>0.05
No	166 (62.6)	99 (37.4)	

* *over previous 12 months; # Chi-square test*.

Comparison revealed that two-thirds of respondents that had consumed and one-third of those that had not consumed OTC drugs had undergone fecal occult blood tests over the previous year (Chi-square test = 6.37, df = 2, *p* < 0.05) and that those respondents had less frequently been hospitalized over the previous 12 months (Chi-square test = 3.67, df = 1, *p* = 0.05) (Table 1).

One-third of OTC drug users who were not able to utilize the required healthcare services, which could be due to a number of factors, such as waiting lists, financial insolvency, or perhaps the distance to the required healthcare institution, were employed. Our research, as well as numerous other studies, showed that fear of losing an existing job could be one of the reasons why employed individuals consume OTC drugs (32).

OTC drug users most often rated their own health as good or average, but despite risky behavior, they had generally never undergone screening for colon cancer. Of those respondents, 56% had chronic health disorders and most often utilized the required form of health care. Most OTC users did not take sick leave and had less frequently been hospitalized or used a day hospital, ambulance, or home treatment in the previous 12 months than had others (Table 1).

**Figure 1:**
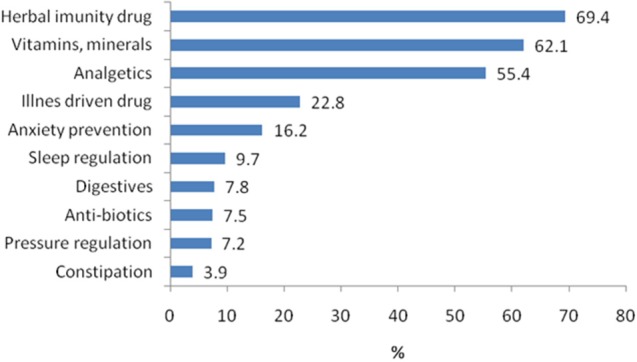
Most commonly consumed drugs, herbal preparations, and vitamins selected by personal choice.

Most common drugs, herbal supplements, and/or vitamins that the respondents were taking on their own, without prior consultation with a physician, were bought in pharmacies and are presented in Figure 1.

Principal component analysis (KMO = 0.715, *p* < 0.01) isolated six factors that explain 53.79% of the variance: Health Condition (17.43% of the variance), Financial Status (10.16% of the variance), Age (7.89% of the variance), OTC Drugs (7.42% of the variance), Risk (6.21% of the variance), and Health Care Utilization (5.94% of the variance).

The health condition factor groups together the variables health self-evaluation, the existence of chronic disease, and limits to daily activities due to the disease, showing that individuals that suffer from chronic diseases that prevent them from undertaking daily activities evaluate their health as bad. The health condition factor included in the factor analysis also implied the presence of chronic disease (Table 2). There was no statistically significant difference in the use of OTC drugs with respect to the presence of a chronic health disorder, such that 64.4% of those who had and 67.3% of those who did not have a chronic illness used OTC products (Chi-square test = 0.29, df = 1, *p* > 0.05).

**Table 2:** Principal component analysis.

**Factor (% of variance)**	**Variables**	**Factor loading**
Health condition (17.43)	Health self-evaluation	−0.759
	Limitations to undertaking daily activities due to illness	0.718
	Chronic disease	0.605
Financial status (10.16)	Accommodation	0.705
	Well-being Index	−0.652
	Education	−0.511
	Region	0.376
	Required visits to health care institutions	0.369
Age (7.89)	Employment	0.546
	Age	0.544
OTC drugs (7.42)	OTC drug use	0.388
	Sex	0.606
	Knowledge about health topics	0.461
	Hospitalization in the previous 12 months	−0.379
Emergency (6.21)	Use of emergency assistance over the previous 12 months	0.428
	Risky behavior	−0.363
Healthcare utilization (5.94)	Availability of selected private practice physician	−0.806
	“Day patient” over the previous 12 months	0.372

Similarly to our research, other studies have shown that the use of certain drugs reduces the rate of hospitalization in adults (33). Antiplatelet, diuretic, and non-steroidal anti-inflammatory drugs and anticoagulants are medicines that reduce hospitalization rates (34).

The financial status factor groups five variables: region, accommodation, well-being index, education, and health care non-utilization. This factor shows that individuals living in urban areas in the Vojvodina area, possessing a higher/high degree of education, and classified as very rich as per the well-being index, have never managed to access required health care services due to long waiting lists.

The age factor groups the variables age and employment status and shows that the oldest respondents were retired.

The OTC drugs factor groups four variables: sex, awareness about health topics, OTC drug use, and hospitalization in the previous 12 months. This factor shows that women who were aware of topics related to health and that had consumed OTC drugs over the previous 12 months had not been hospitalized.

The emergency assistance factor, which groups variables related to using emergency assistance in the previous 12 months and risky behavior, showed that already ill patients had been using emergency medical services during the previous 12 months.

The factor explaining health care utilization groups variables such as the selection of a private practice physician and being a day-patient over the previous 12 months. This shows that respondents who had selected a private practice physician were hospitalized fewer times for additional diagnosis/treatment (“Day patient”) (Table 2).

- Analysis of female respondents aware of health topics via public information systems

The majority of females (71.1%) that followed health topics via public information systems had consumed OTC drugs over the past 2 weeks. Most commonly, they had a high-school diploma. However, 37.2% of females characterized as having a higher/high education level had also used such drugs, while 26.1% of those at the high-school education level had not (Chi-square test = 8.24, df = 2, *p* < 0.05). Every third female in the Vojvodina region had consumed OTC drugs over the previous 2 weeks, while every fourth female from the Sumadija and West Serbia regions had not (Chi-square test = 9.02, df = 3, *p* < 0.05). OTC drugs had been consumed by 56.7% of females following health topics in addition to having risky behavior. Simultaneously, 39.7%, though well-informed on health issues and acting riskily, had not consumed such drugs (Chi-square test = 6.22, df = 2, *p* < 0.05). Further analysis of the female respondents that consumed OTC drugs and followed health-related topics via public information systems (the internet, TV, print, and radio) shows that these respondents had statistically significantly been hospitalized fewer times over the previous year when compared to respondents not using OTC drugs (Chi-square test = 7.98, df = 1, *p* < 0.05). Although those female respondents had not accessed any health care services, there was no significant statistical difference in the usage of OTC drugs among respondents following health topics.

- Analysis of male respondents following health topics via public information systems

Every second respondent (55.3%) following health topics had consumed OTC drugs over the previous 2-week period. The users of OTC drugs were mostly high-school graduates. No significant statistical difference was found among users of OTC drugs that followed health topics and had different levels of education (*p* > 0.05). OTC drugs were mostly used by males living in the Belgrade region (38.6%) and less so by males from the regions of Sumadija and West Serbia (8.8%) (Chi-square test = 8.13, df = 3, *p* < 0.05). No statistically significant difference was found between OTC usage and risky behavior (*p* > 0.05). Among the observed males, no statistically significant difference was found between the use of OTC drugs and hospitalization over the previous 12 months. Of male respondents, 47.8% had neither utilized health care services nor used OTC drugs.

## Discussion

In Serbia, as per our local research, two-thirds (65.3%) of CAM users consume OTC drugs at their own discretion, without either a recommendation from a physician or a prescription. These are mostly females of an average age of 49.16 ± 16.02 with a high-school diploma, married and employed, most frequently living in urban areas, mostly Belgrade, classified as of the middle-income class, and following health topics via public information systems (TV, the internet, radio, and print). Most studies that have been undertaken significantly predict that CAM users will be females and interpret this to be the result of their having more frequent communication with their friends and families about their health condition and having been given diagnoses that turned out to be a significant source of information (23, 35–43). However, there are some studies indicating situations where males turned out to be the majority of consumers in cases where they were suffering from, e.g., gout and arthritis (44).

Our data analyses show that the predominant OTC drug users were females with a high-school diploma and also 37.2% of females with a higher/high education diploma, though 19.2% of such females did not use OTC drugs. Globally, the significant predictors in the majority of studies conducted to date point to CAM users being characterized by a high income and vocations requiring a high level of education. The explanation made for this may lie either in the fact that they were financially able to use CAM or in the fact that CAM users are highly educated and hence question the efficiency of conventional medicine (11, 37–40, 42–45). One study that covered Europe pointed out that the variables of being female and having a higher education level are significant socio-demographic characteristics in CAM usage, while other variables varied depending on the CAM type (36).

Worldwide, health condition and health perception, among selected socio-demographic factors, have been found to be significant in making a decision regarding whether an individual would use CAM and OTC, especially when the condition or the individual's perception of their health was described as bad (34, 36, 42). The OTC users in our research mostly perceived their health as good, i.e., average, and though practicing risky behavior, they had almost never undergone Ca colon cancer tests. Fifty-six percent of these types of respondents suffer from chronic diseases and mostly utilized the required health care services. Worldwide, as per different studies, patients suffering from chronic diseases or some form of disability (35) and other more challenging types of disease (40, 46, 47), decided to use CAM and OTC drugs very frequently due to a lack of expected effects from conventional medicinal treatments or due to the non-availability of a General Practitioner (36). A systematic review of 18 studies in nine countries observing the use of CAM in the diabetic patient population, where prevalence varies from 17 to 72% (average 45%), determined that dietetic supplements, herbal medicines, nutrition consultations, spiritualism, and relaxing techniques were the methods most frequently applied (46). A review of six studies in Saudi Arabia showed the prevalence of CAM use among diabetic patients to be 32.18% and that herbal medicines were most commonly used (47). The most common types of medicines in our research were herbal medicines and/or vitamins that were consumed by the patients on their own, without previous consultation with a physician, and were bought in pharmacies.

In Serbia, according to the Medicines and Medical Devices Act, the advertisement of medicines and medical devices is prohibited, that is, the information that is advertised on a medicinal product or medical device must be true and scientifically proven, cannot mislead the professional and general public, and must be in accordance with the Regulation on the Advertising of Medicines and Medical Devices (48). However, advertising is allowed for OTC drugs. Based on research conducted among students in Poland, we learned that the advertising of OTC drugs is allowed there and is on the rise (49). Interestingly, in both Poland and our research, the largest portion of OTC consumers were females (49). The statistics show that females in Serbia follow health-related topics via the media (TV, the internet, radio, and print), like the females in Poland, but that females were more attentive and less influenced by advertising (49).

Thanks to the research undertaken in Serbia (January 2010–July 2011), pioneering efforts have been made that have resulted in an understanding of the weaknesses in the perceptions of the current and future health workforce toward CAM. It included a questionnaire based on the existing CAM Health Belief Questionnaire (CHBQ), using the Likert scale, where 797 health students and practitioners were interviewed. Students of Dentistry (54.65 ± 6.07) were better informed on CAM therapies than students of Medical Science (50.26 ± 7.92) and students of Pharmacy Science (51.16 ± 7.10). Physicians achieved better results than university professors (55.12 ± 6.55 vs. 50.29 ± 9.50). Furthermore, all of the respondents gave priority to the use of vitamins over any other CAM therapy. In general, consciousness of the use of CAM is increasing in the Balkans (50).

Systematic literature review (42 studies) has found that the prevalence of CAM use ranged from 8 to 90% in patients with prostate cancer (39). A systematic review of four studies on the population of patients with colorectal cancer showed that up to 75% of them used at least one CAM method (51). The most commonly used CAM methods were biologically based therapies: herbal remedies (48.7%), homeopathy (20.5%), vitamins/minerals (17.9%), medical teas (15.4%), and mind and body medicinal procedures such as spiritual techniques (15.4%) and relaxation techniques (12.8%) (51). According to the results of a systematic review of 49 studies on the use of CAM in patients with arthritis in 12 countries, dietary supplements and massage were the most frequently used (43). For Parkinson's patients, the estimated prevalence of CAM use was found to range from 25.7 to 76%, with the most commonly used CAM methods being massage in the United Kingdom, acupuncture in Sweden and Argentina, vitamins and herbal remedies in the United States, traditional medicine in Singapore, and oriental medicine in South Korea (44).

## Conclusion

The analysis of socio-demographic factors and their influence on the proper selection of OTC drugs should become a very significant part of the processes conducted in order to define strategies for determining the treatments for particular patients. The research statistics presented here strongly support OTC drugs correlates with an absence of hospitalization, absence of medical leaves, and absence of day-care hospital, emergency assistance, or home medical care usage in the previous 12 months. Furthermore, wider research focusing on the aforementioned factors should enable the improvement of treatment strategies for enhancing patients' well-being.

In addition, although global research indicates growing trends, the actual evidence for the use of CAM methods in Serbia is very poor. In order to enable a comparison of health care service utilization and CAM methods, it would be necessary to take a different approach. This new path would rely on the Ministry of Health of the Republic of Serbia taking a leading role, due to their better understanding of this matter.

## Data Availability Statement

Publicly available datasets were analyzed in this study. This data can be found here: https://ec.europa.eu/health/indicators_data/indicators_el; https://ec.europa.eu/health/indicators_data/echi_en; http://www.healthpowerhouse.com.

## Author Contributions

ML-S and MJ jointly designed the study and defined the research questions. NM and NR did most of the data mining and extraction, purification of files for missing data and artifacts, and statistical analysis. NM, NR, and ML-S contributed to table and figure creation and interpretation of data. ML-S, MJ, and NR drafted the working version manuscript. All authors contributed to the final version to the extent of important intellectual content.

### Conflict of Interest

The authors declare that the research was conducted in the absence of any commercial or financial relationships that could be construed as a potential conflict of interest.
